# Portrayals of mental illness, treatment, and relapse and their effects on the stigma of mental illness: Population-based, randomized survey experiment in rural Uganda

**DOI:** 10.1371/journal.pmed.1002908

**Published:** 2019-09-20

**Authors:** Justin D. Rasmussen, Bernard Kakuhikire, Charles Baguma, Scholastic Ashaba, Christine E. Cooper-Vince, Jessica M. Perkins, David R. Bangsberg, Alexander C. Tsai

**Affiliations:** 1 Massachusetts General Hospital, Boston, Massachusetts, United States of America; 2 Duke University, Durham, North Carolina, United States of America; 3 Mbarara University of Science and Technology, Mbarara, Uganda; 4 University of Geneva, Geneva, Switzerland; 5 Peabody College, Vanderbilt University, Nashville, Tennessee, United States of America; 6 Oregon Health Sciences University–Portland State University School of Public Health, Portland, Oregon, United States of America; 7 Harvard Medical School, Boston, Massachusetts, United States of America; Addis Ababa University / King's College London, ETHIOPIA

## Abstract

**Background:**

Mental illness stigma is a fundamental barrier to improving mental health worldwide, but little is known about how to durably reduce it. Understanding of mental illness as a treatable medical condition may influence stigmatizing beliefs, but available evidence to inform this hypothesis has been derived solely from high-income countries. We embedded a randomized survey experiment within a whole-population cohort study in rural southwestern Uganda to assess the extent to which portrayals of mental illness treatment effectiveness influence personal beliefs and perceived norms about mental illness and about persons with mental illness.

**Methods and findings:**

Study participants were randomly assigned to receive a vignette describing a typical woman (control condition) or one of nine variants describing a different symptom presentation (suggestive of schizophrenia, bipolar, or major depression) and treatment course (no treatment, treatment with remission, or treatment with remission followed by subsequent relapse). Participants then answered questions about personal beliefs and perceived norms in three domains of stigma: willingness to have the woman marry into their family, belief that she is receiving divine punishment, and belief that she brings shame on her family. We used multivariable Poisson and ordered logit regression models to estimate the causal effect of vignette treatment assignment on each stigma-related outcome. Of the participants randomized, 1,355 were successfully interviewed (76%) from November 2016 to June 2018. Roughly half of respondents were women (56%), half had completed primary school (57%), and two-thirds were married or cohabiting (64%). The mean age was 42 years. Across all types of mental illness and treatment scenarios, relative to the control vignette (22%–30%), substantially more study participants believed the woman in the vignette was receiving divine punishment (31%–54%) or believed she brought shame on her family (51%–73%), and most were unwilling to have her marry into their families (80%–88%). In multivariable Poisson regression models, vignette portrayals of untreated mental illness, relative to the control condition, increased the risk that study participants endorsed stigmatizing personal beliefs about mental illness and about persons with mental illness, irrespective of mental illness type (adjusted risk ratios [ARRs] varied from 1.7–3.1, all *p* < 0.001). Portrayals of effectively treated mental illness or treatment followed by subsequent relapse also increased the risk of responses indicating stigmatizing personal beliefs relative to control (ARRs varied from 1.5–3.0, all *p* < 0.001). The magnitudes of the estimates suggested that portrayals of initially effective treatment (whether followed by relapse or not) had little moderating influence on stigmatizing responses relative to vignettes portraying untreated mental illness. Responses to questions about perceived norms followed similar patterns. The primary limitations of this study are that the vignettes may have omitted context that could have influenced stigma and that generalizability beyond rural Uganda may be limited.

**Conclusions:**

In a population-based, randomized survey experiment conducted in rural southwestern Uganda, portrayals of effectively treated mental illness did not appear to reduce endorsement of stigmatizing beliefs about mental illness or about persons with mental illness. These findings run counter to evidence from the United States. Further research is necessary to understand the relationship between mental illness treatment and stigmatizing attitudes in Uganda and other countries worldwide.

**Trial registration:**

The experimental procedures for this study were registered with ClinicalTrials.gov as "Measuring Beliefs and Norms About Persons With Mental Illness" (NCT03656770).

## Abstract 2

Justin Rasmussen and colleagues study reactions to representations of mental illness in Ugandan women.

## Author summary

**Why was this study done?:**

- Mental illness stigma is a fundamental barrier to improving mental health worldwide.
- While there has been some progress in understanding how to reduce mental illness stigma in high-income countries, it is unclear how this understanding might generalize to low- and middle-income countries.
- The extent to which people perceive that mental illness can be effectively treated may be an important component of changing negative beliefs about mental illness.

**What did the researchers do and find?:**

- We conducted a survey experiment to understand how information about successful treatment of mental illness might affect stigmatizing beliefs in rural southwestern Uganda.
- This experiment involved randomly assigning different people in eight villages to be read a vignette about: a woman who had signs suggestive of one of three different types of mental illness; a woman who had these signs and was treated successfully; or a woman who had these signs and was treated successfully but subsequently relapsed.
- We found that stigma toward mental illness in the community was common and was generally unaffected by descriptions of successful treatment.

**What do these findings mean?:**

- If unaddressed, stigma will continue to pose a major barrier to improving population mental health in Uganda.
- We need to do more research to understand the relationship between perceptions of mental illness treatment and stigmatizing attitudes in Uganda and other countries worldwide.
- Engaging local etiologies, making treatment more accessible, and understanding how mental illness shapes social relationships independent of actual symptoms might be important avenues of research and program implementation to explore.

## Introduction

Mental illness is heavily stigmatized worldwide. In cross-national studies, people with mental illness report experiencing discrimination in most areas of their life, including making friends, keeping jobs, or interacting with their partners and families [1–3]. Available evidence suggests that while beliefs about mental illness vary by country, negative attitudes toward people with mental illness are neither uncommon nor isolated [4,5]. Widespread negative attitudes provide an enabling environment for harmful violations of basic human rights that range in severity from prejudicial behavior and employment discrimination to chaining, caging, and killing [6,7]. These attitudes undercut efforts to improve mental health at a fundamental level because stigma undermines already low rates of mental-healthcare–seeking behavior [8–18]. Compounding this attenuating effect on treatment-seeking, stigma is also associated with reduced public support for funding toward mental health services, which erodes the availability of appropriate care within the mental healthcare system [19–21].

Attempts to reduce stigma in high-income countries have achieved some measure of success, though the durability of these results is uncertain [22,23]. Furthermore, the evidence, especially from low- and middle-income countries (LMICs), is not sufficient to understand the extent to which these interventions can be generalized [24–32]. Contrary to the hypotheses that motivated many of the early large-scale awareness campaigns in many high-income countries during the 1990s and 2000s, increasing understanding of the potential biological underpinnings of mental illness has not positively influenced attitudes toward persons with mental illness, and there are some circumstances in which this increasing biological understanding may have even worsened stigma [26,33–38]. The successes of recent contact-based interventions that promote meaningful contact with persons with mental illness alongside targeted education appear to hold some promise for long-term stigma reduction efforts [22,23,39–42]. While results from this class of interventions are somewhat mixed, a key ingredient of successful contact interventions seems to be an emphasis on recovery [43,44]. It may be, then, that one reason early interventions failed to reduce stigma is that the stigma attached to mental illness is driven in large part by beliefs about the extent to which it can be treated [45], and, despite increasing acceptance of biological causes of mental illness, many people continue to perceive mental illness to be untreatable [10,46].

Experiments that have been conducted to examine the relationship of treatment information to stigmatizing attitudes in the United States have shown that providing study participants with vignettes describing successful treatment of mental illness can reduce desire for social distance and negative attitudes about mental illness and can enhance beliefs in the effectiveness of treatment [47–49]. This treatable-illness hypothesis has not been tested internationally, but related literature on HIV stigma in sub-Saharan Africa suggests that the widespread availability of effective HIV antiretroviral treatment has enabled people with HIV to actively contribute (socially and economically) to their communities, thereby reducing internalized stigma among people with HIV and enhancing their standing in the general community [50–56]. Taken together, these studies suggest that the connection between perceptions of treatability and mental illness stigma may also generalize to contexts like sub-Saharan Africa.

To investigate the treatable-illness hypothesis, we embedded a survey experiment into a whole-population cohort study in rural southwestern Uganda. Adapting vignettes from the General Social Survey and the novel study by McGinty and colleagues [48], we aimed to determine whether the extent to which mental illness was portrayed as a treatable medical condition affected personal beliefs and perceived norms about mental illness and about people with mental illness. Specifically, we hypothesized that descriptions of mental illness alone and relapse after initially effective treatment would elicit more stigmatizing responses compared with descriptions of successfully treated mental illness.

## Methods

### Study population

This study was conducted in the eight villages of Nyakabare Parish, a rural administrative subunit of Mbarara District in the southwest region of Uganda. The study site is representative of rural communities in Mbarara District; it is relatively isolated, with an economy dominated by small-scale farming, animal husbandry, and petty trading, and both food and water insecurity are common [57,58]. All procedures were embedded within an ongoing, whole-population social network cohort study in which study participants are surveyed biennially [59]. The study includes all adults aged 18 years and above (and emancipated minors aged 16–17 years) who maintain stable primary residence within the roughly 11-square-kilometer area of the parish and who can give informed consent to participate. Exclusions include people who cannot communicate meaningfully with research staff, for example, because of deafness, mutism, or aphasia; people with behavioral problems thought to represent psychosis, neurological damage, or acute intoxication; and people too cognitively impaired to provide informed consent. Participants in the baseline survey, which was completed in 2015, served as the randomization sample for the present survey experiment. The second biennial survey was fielded between November 2016 and June 2018, providing the data reported in this manuscript.

### Experimental design and data collection

A total of 1,776 participants (who were enumerated in the 2014–2015 baseline survey) were randomly assigned to receive one of 10 different vignettes describing a young woman (see **S1 Text**). Reporting was guided by the CONSORT checklist (see **S1 CONSORT checklist**). The control vignette described the demographic characteristics and basic life story of a typical Ugandan woman with no further elaboration. The remaining nine vignettes included the same basic description of the woman but also described her experiencing three different types of symptoms (psychosis, mania, and depression, suggestive of schizophrenia, bipolar disorder, and major depressive disorder, respectively), each with three different treatment outcomes (no treatment, successful treatment followed by recovery, and successful treatment followed by recovery and then relapse/recurrence). These vignettes were adapted from McGinty and colleagues [48] to fit the local context based on feedback from key informants, documented symptom presentation in Uganda, and consultation with psychiatrists at the Mbarara Regional Referral Hospital.

Each eligible person was approached in the field, typically at their home or place of employment, by a research assistant who spoke the local language (Runyankore) and who requested their participation in the study. The survey was framed in general terms as a study about the social lives and health of residents of Nyakabare Parish, not as a study about beliefs about mental illness. For persons who expressed potential interest, the study was described in detail, and their written informed consent to participate was obtained. Study participants who could not sign their name were permitted to indicate consent with a thumbprint. All research assistants received in-depth training on how to administer surveys for gathering sensitive information, including instructions on how to temporarily halt the survey if another person came within earshot.

After study participants were presented with one of the 10 vignettes, they were asked to respond to three questions regarding their personal beliefs about mental illness and about people with mental illness, and three questions regarding their perceptions of village norms about mental illness and about people with mental illness. (For the sake of parsimony in writing, hereafter we refer to the subject of these questions using the shorthand “about mental illness.”) Specifically, participants were asked whether they would allow the woman to marry a member of their family, whether they believed the woman was receiving divine punishment for engaging in immoral behavior, and whether they believed she brought shame upon her family. These questions measure three different domains of stigma that are salient to the local context: social distance [60–62], etiological attribution [34], and courtesy stigma [63–65]. In response to the three items about personal beliefs, participants were allowed to provide one of five different responses: “Yes,” “No,” “It depends on knowing more details,” “Do not know,” and “Refuse to respond.”

Paralleling these three outcome variables measuring personal beliefs, study participants were also asked about the proportion of other people in their village who would allow the woman to marry a member of their families, the proportion of other people in the village who believed the woman was receiving divine punishment for engaging in immoral behavior, and the proportion of other people in their village who believed she brought shame upon her family. These questions measure the same domains of stigma (social distance, etiological attribution, and courtesy stigma) but focus on perceived norms rather than personal beliefs. These questions were modeled after previously published studies of perceived norms about different health behaviors and health risk behaviors [66–69]. The survey questions specified “your village” so that all participants would have a similar fixed, unambiguous reference group when describing their perceptions about the norm within their villages [70]. Response options for the items about perceived norms followed a four-point Likert-type scale (in addition to “Do not know” and “Refuse to respond”): “All or almost all,” “More than half,” “Fewer than half,” and “Very few, or no one.”

The English translations of the six outcome measures are provided in **S1 Text**. The vignettes and associated survey questions were first written in English, translated into Runyankore, and then back-translated into English to verify the fidelity of the translated text. The translation and back-translation followed an iterative process involving in-depth consultation and pilot testing with key informants.

Participants were allocated to the 10 vignettes in equal proportions in a parallel group design in which treatment assignment was determined centrally using a computerized random number generator. Neither the research assistants administering the questionnaires nor the study participants were aware of the vignettes to which the study participants had been assigned. The research assistants were not blinded, however, so they likely perceived the differences in the vignettes being administered to different study participants. To ensure balance across sex and village strata, we generated 16 separate randomization schedules for subsets of participants defined by strata of sex and village of residence [71]. The vignettes and all associated survey questions were programmed into laptop computers running the Computer Assisted Survey Information Collection (CASIC) Builder software program (West Portal Software Corporation, San Francisco, CA, USA) so that the surveys could be administered in the field. The experimental procedures for this study were registered with ClinicalTrials.gov (NCT03656770). The protocol record was entered in April 2017 but, because of an administrative error, was not released on ClinicalTrials.gov until August 2018.

### Analysis

The analysis was prespecified prior to data collection (**S2 Text**). For the three outcome variables measuring personal beliefs, responses were coded such that 0 denoted a nonstigmatizing response and 1 denoted a stigmatizing or ambivalent response. Namely, unwillingness to allow the woman to marry a member of the participant’s family, belief that she was receiving divine punishment, and belief that she brought shame on her family were assigned values of 1, along with the ambivalent response of “it depends.” Willingness to allow the woman to marry into the family, belief that she was not receiving divine punishment, and belief that she did not bring shame upon her family were assigned values of 0. “Do not know” and “refuse” were considered missing data. We then fitted Poisson regression models specifying each outcome as the dependent variable and the vignette treatment assignment as the primary exposure of interest. Cluster-correlated robust estimates of variance were used so that the estimated incidence rate ratios could be interpreted as risk ratios [72,73].

For the three 4-level categorical outcome variables measuring perceived norms, responses were coded 1–4 such that the lowest category denoted the least perceived stigma and the highest category denoted the most perceived stigma. (For example, in response to the question about whether others in the village would permit the woman in the vignette to marry into the family, “Very few, or no one” was the highest category and “All or almost all” was the lowest category.) “Do not know” and “refuse” were considered missing data. We then fitted ordinal logit regression models specifying each outcome as the dependent variable and the vignette treatment assignment as the primary exposure of interest. The exponentiated regression coefficients were interpreted as estimated odds ratios. To confirm that the regression coefficients did not vary across the logit equations (i.e., the assumption of proportional odds), we used the omnibus Wald test by Brant [74].

To ensure accurate confidence intervals that accounted for the stratified randomization scheme, we adjusted treatment estimates for sex and village of residence by including them as covariates in the respective Poisson and ordinal logit regression models described above. Stata statistical software was used to conduct all data cleaning and analysis (version 14.0, College Station, TX, USA).

### Ethics statement

Ethical approval for this study was granted by the Partners Human Research Committee at Massachusetts General Hospital and the Research Ethics Committee at Mbarara University of Science and Technology. We also received clearance for the study from the Uganda National Council of Science and Technology and the Research Secretariat in the Office of the President of the Republic of Uganda.

## Results

Of the 1,776 participants enumerated and randomized in the 2014–2015 survey, 1,355 (76%) were successfully interviewed in 2016–2018, excluding 10 individuals who were not administered the experiment correctly because of a technical error. Of the remainder, 250 (14%) were known to have emigrated out of the study site, 57 (3%) could not be located, 37 (2%) had died, 42 (2%) refused to participate, and 25 (1%) were ineligible or could not be interviewed for other reasons (for example, incarceration or acute intoxication at each of multiple interview attempts). We summarize participant characteristics in **Table 1**. Respondents came from all eight villages, and just over half were women (56%). The mean age was 42 years, with good representation from all age groups. Just over half (57%) had completed primary school, and almost two-thirds were married or cohabiting (64%).

**Table 1:** Participant characteristics.

	N	%
Sex		
Male	599	44%
Female	756	56%
Education		
None	197	15%
Some primary (P1–P6)	393	29%
Completed primary (P7)	332	25%
Beyond primary (S1–S6, vocational, university)	433	32%
Marital status		
Single	484	36%
Married/cohabiting	871	64%
Age category		
18–25 years	209	15%
26–35 years	337	25%
36–45 years	274	20%
46–55 years	248	18%
56+ years	263	19%
Unknown	24	2%
Village		
1	210	15%
2	192	14%
3	177	13%
4	156	12%
5	110	8%
6	202	15%
7	112	8%
8	196	14%

A technical error in survey administration resulted in some treatment assignments that departed from the intended randomization. (**S3 Text** provides further detail about the nature of the error, a comparison of the correctly versus incorrectly assigned participants, and results of a sensitivity analysis based on a data set excluding the incorrectly assigned participants. As shown in the S3 Text, neither the reported results nor final conclusions were substantively affected by the error.) Participants who received a vignette portraying any kind of mental illness reported more stigmatizing personal beliefs compared with those who received the control vignette, across all outcomes, for every variant of symptom presentation, and for every variant of treatment description (**Table 2**). Across outcomes, relative to the control vignette (22%–30%), substantially more study participants believed the woman in the vignette was receiving divine punishment (31%–54%) or believed she brought shame on her family (51%–73%), and most were unwilling to have her marry into their family (80%–88%). A small number of study participants provided ambivalent responses (1%–8%, depending on the outcome), and there were negligible refusals (<1%) and "don't know" (0%–4%) responses.

**Table 2:** Stigmatizing personal beliefs by treatment assignment.

Stigmatizing Personal Beliefs		Unwilling for family member to marry^a^	Is receiving divine punishment^a^	Brings shame on family^a^
Control		37 (30%)	27 (22%)	29 (23%)
Schizophrenia	Mental illness	120 (86%)	58 (41%)	90 (64%)
	+ Treatment	120 (86%)	56 (40%)	72 (51%)
	+ Relapse	249 (83%)	108 (36%)	170 (56%)
Bipolar	Mental illness	116 (87%)	72 (54%)	98 (73%)
	+ Treatment	98 (78%)	50 (40%)	68 (54%)
	+ Relapse	71 (80%)	29 (33%)	49 (55%)
Depression	Mental illness	82 (80%)	36 (35%)	62 (61%)
	+ Treatment	80 (78%)	40 (39%)	55 (54%)
	+ Relapse	86 (88%)	30 (31%)	50 (51%)

^a^N (%) refer to the number and proportion of study participants assigned to each treatment arm who endorsed the stigmatizing belief shown in the column header. Column percentages do not add to 100% because each column represents a different outcome variable (i.e., the columns do not represent categories of a single categorical variable).

Compared with the responses to questions about their personal beliefs, study participants’ responses to questions about perceived norms about people with mental illness followed similar patterns, though the differences in comparison with the control vignette were not as large in magnitude (**Table 3**). Once again, there were negligible refusals (<1%) and "don't know" (0%–6%) responses.

**Table 3:** Perceived stigmatizing beliefs of others by treatment assignment.

Perception that Most Others (>50% of Others) Hold Stigmatizing Belief^a^		Most others unwilling for family member to marry^b^	Most others believe receiving divine punishment^b^	Most others believe Brings shame on family^b^
Control		38 (30%)	25 (20%)	23 (18%)
Schizophrenia	Mental illness	114 (81%)	39 (28%)	59 (42%)
	+ Treatment	113 (81%)	47 (34%)	62 (44%)
	+ Relapse	257 (86%)	79 (26%)	126 (42%)
Bipolar	Mental illness	117 (87%)	50 (37%)	67 (50%)
	+ Treatment	104 (83%)	31 (25%)	51 (41%)
	+ Relapse	75 (84%)	21 (24%)	37 (42%)
Depression	Mental illness	76 (75%)	32 (31%)	41 (40%)
	+ Treatment	86 (84%)	33 (32%)	36 (35%)
	+ Relapse	83 (85%)	21 (21%)	42 (43%)

^a^The numbers and percentages in each cell refer to the percentage of study participants who believe that most others (>50% of others) in their village hold the stigmatizing belief in question.

^b^N (%) refer to the number and proportion of study participants assigned to each treatment arm who endorsed the stigmatizing belief shown in the column header. Column percentages do not add to 100% because each column represents a different outcome variable (i.e., the columns do not represent categories of a single categorical variable).

Using Poisson regression models that also adjusted for the stratification variables, we found that portrayals of mental illness significantly increased the risk of stigmatizing responses compared to the control vignettes, across all outcomes, for every variant of symptom presentation, and for every variant of treatment description—except one (**Table 4**). Namely, participants who received the vignette describing a woman receiving effective treatment for depressive symptoms but then experiencing a subsequent relapse were no more likely to believe she was receiving divine punishment than participants in the control group (adjusted relative risk [ARR] = 1.46, 95% CI 0.89–2.51, *p* = 0.18). Apart from that exception, participants who received vignettes depicting a woman with a mental illness were, depending on the symptom presentation and treatment experience described, 2.64–2.98 times more likely (than those who received the control vignette) to be unwilling to allow a family member to marry her, 2.20–3.14 times more likely to believe that she brought shame upon her family, and 1.53–2.49 times more likely to believe that she was receiving divine punishment (all *p*-values < 0.05).

**Table 4:** Risk of stigmatizing personal beliefs by treatment assignment based on Poisson regression.

Stigmatizing Personal Beliefs		Unwilling for family member to marry		Is receiving divine punishment		Brings shame on family	
		ARR (95% CI)	*p*-value	ARR (95% CI)	*p*-value	ARR (95% CI)	*p*-value
Control		Reference		Reference		Reference	
Schizophrenia	Mental illness	2.9 (2.0–4.2)	<0.001	2.0 (1.3–2.9)	0.001	2.8 (2.0–3.8)	<0.001
	+ Treatment	2.9 (1.9–4.4)	<0.001	1.9 (1.0–3.3)	0.035	2.2 (1.4–3.6)	0.001
	+ Relapse	2.8 (1.9–4.2)	<0.001	1.7 (1.1–2.7)	0.020	2.5 (1.8–3.5)	<0.001
Bipolar	Mental illness	3.0 (1.9–4.5)	<0.001	2.5 (1.9–3.3)	<0.001	3.1 (2.2–4.4)	<0.001
	+ Treatment	2.6 (1.9–3.6)	<0.001	1.8 (1.3–2.6)	<0.001	2.4 (1.7–3.3)	<0.001
	+ Relapse	2.7 (1.8–4.1)	<0.001	1.5 (1.0–2.3)	0.03	2.4 (1.8–3.2)	<0.001
Depression	Mental illness	2.7 (1.8–4.1)	<0.001	1.7 (1.2–2.3)	0.002	2.6 (1.8–3.7)	<0.001
	+ Treatment	2.7 (1.8–4.1)	<0.001	1.8 (1.1–3.1)	0.027	2.3 (1.6–3.5)	<0.001
	+ Relapse	3.0 (2.0–4.5)	<0.001	1.5 (0.8–2.5)	0.177	2.2 (1.5–3.2)	<0.001

**Abbreviations:** ARR, adjusted risk ratio.

Contrary to our hypotheses motivated by the work of McGinty and colleagues [48], study participants who received vignettes depicting effective treatment were only slightly less likely to endorse stigmatizing responses compared with those who received vignettes depicting untreated mental illness. In the case of bipolar illness, participants receiving the vignette about effective treatment were still more likely to endorse stigmatizing responses than participants receiving the control vignette (ARRs ranged from 1.8–2.6, all *p* < 0.001), but the ARRs were smaller in magnitude compared with those receiving the vignette about untreated bipolar illness (ARRs ranged from 2.5–3.1, all *p* < 0.001) (*p*-value for comparisons all <0.05).

Results from the ordered logit regression models comparing perceived norms followed a similar pattern to that of personal beliefs (**Table 5**). Across all outcomes, for every variant of symptom presentation, and for every variant of treatment description, a vignette describing any type of mental illness (untreated, treated, or treated with relapse) increased the odds that participants perceived more people in their village would be unwilling to allow the woman to marry into their families, participants perceived more people would believe that she brings shame upon her family, and participants perceived more people would believe that she was receiving divine punishment. As with the data on personal beliefs, in the case of bipolar illness, participants receiving the vignette about effective treatment were still more likely to endorse stigmatizing responses than participants receiving the control vignette (adjusted odds ratios [AORs] ranged from 1.8–10.5, all *p* < 0.001) but the AORs were smaller in magnitude compared with those receiving the vignette about untreated bipolar illness (AORs ranged from 3.1–16.3, all *p* < 0.001) (*p*-value for comparisons all <0.01).

**Table 5:** Odds of perceiving stigmatizing beliefs of others by treatment assignment based on ordered logit regression.

Perceived Norms		Most others unwilling for family member to marry		Most others believe receiving divine punishment		Most others believe brings shame on family	
		AOR (95% CI)	*p*-value	AOR (95% CI)	*p*-value	AOR (95% CI)	*p*-value
Control	Reference						
Schizophrenia	Mental illness	14.0 (6.3–31.4)	<0.001	2.1 (1.5–2.9)	<0.001	3.8 (2.0–7.1)	<0.001
	+ Treatment	9.2 (3.5–24.3)	<0.001	2.7 (1.6–4.6)	<0.001	3.9 (1.9–7.9)	<0.001
	+ Relapse	15.7 (7.8–31.7)	<0.001	2.0 (1.4–2.7)	<0.001	3.6 (2.5–5.1)	<0.001
Bipolar	Mental illness	16.3 (7.0–37.8)	<0.001	3.1 (2.1–4.6)	<0.001	5.1 (4.0–6.4)	<0.001
	+ Treatment	10.5 (4.7–23.7)	<0.001	1.8 (1.4–2.5)	<0.001	3.3 (2.5–4.5)	<0.001
	+ Relapse	9.1 (4.1–20.2)	<0.001	2.0 (1.2–3.3)	0.008	3.1 (1.8–5.2)	<0.001
Depression	Mental illness	6.9 (2.5–19.0)	<0.001	2.8 (1.8–4.3)	<0.001	3.3 (1.9–5.9)	<0.001
	+ Treatment	11.0 (4.5–26.9)	<0.001	2.6 (1.8–3.8)	<0.001	3.1 (1.6–6.0)	0.001
	+ Relapse	11.7 (5.4–25.3)	<0.001	1.7 (1.1–2.5)	0.012	3.6 (2.3–5.4)	<0.001

The estimated AORs in each cell refer to the relative odds of being in a higher category of perceiving that more people in their village (ranging from “very few, or no one” to “all or almost all”) hold the stigmatizing belief in question. **Abbreviations:** AOR, adjusted odds ratio.

## Discussion

In this population-based, randomized survey experiment conducted in rural southwestern Uganda, portrayals of effectively treated mental illness did not appear to reduce endorsement of stigmatizing responses about mental illness. Instead, any kind of mental illness portrayal—whether untreated, successfully treated, or treated with relapse—resulted in an overwhelmingly large proportion of stigmatizing responses. Among those responses, refusal to have a woman with mental illness marry into the family was the most common, though beliefs that her mental illness created shame for the family or was the result of divine punishment were also fairly common. Perceptions of village norms followed similar patterns as individual beliefs.

Our primary finding that varying degrees of treatment success had no ameliorating effect on stigmatizing beliefs runs contrary to other similar studies conducted in the United States, which have found that portrayals of effective treatment reduced stigma for a variety of mental illnesses [47–49]. Below, we offer several possible reasons why treatment information may have produced differing levels of stigmatizing responses in this study as compared with previously published work.

Lebowitz and Ahn’s [47] experiment examining the role of etiology in stigma and treatment descriptions may provide one explanation for this discrepancy. By varying the description of mental illness etiology as well its treatment, they showed that attributing a mental illness to biological origins largely parallels the findings of other studies, with worsened stigma resulting when the vignette did not mention treatment but reduced stigma resulting when the vignette described effective treatment. By contrast, ascribing a nonbiological cause to the mental illness rendered the treatment information irrelevant. In our study, we may have observed this latter effect. Nonbiological interpretations of mental illness are widely held in Uganda [75–79] and sub-Saharan Africa generally (particularly in rural areas) [80–85], unlike many high-income countries, which have experienced steady shifts over the past several decades in the direction of a neurobiological understanding of mental illness [34,86]. One potential interpretation of our findings is that participants may have viewed the symptoms as essentially nonbiological and therefore received the information about biomedical treatment as being less salient (compared with participants in studies previously conducted in the United States). What we described as “effective treatment” may not have addressed what many of our participants understood to be the true ailment and source of stigma [87].

Apart from etiology, confidence in the treatment process itself may also explain the discrepant findings. Uganda’s mental health system, much like those in other low-income countries, lacks the resources necessary to consistently provide effective treatment across the country, with only 2.96 mental health workers (including just 0.09 psychiatrists and 2.24 mental health nurses) per 100,000 people [88]. With pharmaceutical treatment availability concentrated in urban areas and typically limited to older, cheaper, and less effective medications, participants in this study may have perceived mental health treatment to be largely ineffective or inaccessible [76,77,89]. These perceptions may have attenuated the effect that providing them with treatment information could have had on stigmatizing beliefs elicited in the survey [90].

Even with descriptions of etiologically appropriate or available treatment, participants may still have understood mental illness to be essentially permanent. Key informants pointed to a local Runyankore proverb, “one who has been mad will always scare the children,” that captures the widely held perception that mental illness is simply not treatable. This belief that mental illness is never truly eliminated even if treated to long-term remission likely interacts in significant ways with etiological attributions and experiences with the mental healthcare system. However, as Schnittker and colleagues [46] note in their reflection on the failures of genetic descriptions to reduce stigma in the US, etiological beliefs and even endorsement of treatment are distinct from the idea that a person can truly recover from mental illness. This belief that mental illness is a permanent condition independent of its symptoms is most characteristic of classic stigma in that it is a “mark” or label that relates people with mental illness to undesirable attributes intrinsically as a permanent identity [65,91,92]. The loss of status associated with this label, as the results of our study show, might never be reversed even if someone resumes all roles and social functions symptom-free. Many of the studies on mental illness and its associated stigma in Uganda, and in sub-Saharan Africa generally, have focused on causation and explanatory models [75–85]. There has been far less attention to the ways mental illness can shape enduring social identities and relationships that may be only indirectly related to actual symptoms and treatment [93,94].

A second important finding of our study is that we observed highly stigmatizing responses regardless of symptom presentation and across multiple domains. Key informant feedback suggests that participants in our study did not differentiate between the different symptom presentations and instead likely understood all variations in symptom presentation to represent a single category of mental illness. Whether or not these anecdotal observations held true throughout the study sample, our data show that stigma toward people exhibiting symptoms of mental illness was very common, consistent with the pattern of findings documented in other studies [1–3]. That most participants in our study believe mental illness brings shame on a person’s family also highlights the effects stigma can have on isolating people and undermining their ability to obtain social support [64,95–97].

Interpretation of our findings is subject to several important limitations. Our use of vignettes as the primary experimental manipulation allowed us to vary key details of each description. Nonetheless, study participants were only exposed to hypothetical scenarios that were potentially lacking in context that might influence stigma [62,98]. This limitation is especially important in light of evidence that the stigma of mental illness can interact and be compounded by other intersecting identities [99]. For example, several studies have shown that gender can interact with mental illness type to affect stigma [100,101], and recent evidence from related literature suggests that socioeconomic status may also influence participant responses [102]. Since our vignettes only described a woman with average socioeconomic status for the region, we are unable to determine whether and how descriptions of other identities could have yielded differences in stigmatizing responses. For example, if the vignettes had portrayed a similar man with mental illness, it is possible that the findings would have been different. Second, it is possible that social desirability bias could have affected study participants’ responses [103]. If negative attitudes toward persons with mental illness were understood to be socially undesirable, study participants might have overestimated the proportion of others who would endorse stigmatizing attitudes while underreporting their own stigmatizing attitudes toward persons with mental illness [62,103,104]. This phenomenon was not observed in the data. Participants generally overestimated the proportion of others who would be unwilling to have the woman marry into their families but generally underestimated the extent to which others believed the woman was receiving divine punishment or brought shame on her family. It is important to note that, while social desirability bias could have affected the overall levels of stigma in the population, it is an unlikely explanation for the differences in levels of stigma across the treatment arms (given the randomized design). A third limitation is that this experiment focused on mental illnesses but did not include descriptions of substance use disorders, for which perceptions and attitudes are likely to differ significantly [48,105–107]. Fourth, our findings may not generalize beyond this rural region of southwestern Uganda. Other studies have found important differences in mental illness stigma between rural and urban areas [108,109]. However, the study was based on a whole-population sample, and the community we surveyed shares important characteristics with the rest of the country and the East African region. Finally, it is important to note that a brief vignette portrayal of mental illness as a treatable health condition might not have an enduring educational effect (that could therefore translate into an enduring antistigma effect). It is possible that more sustained education about the treatability of mental illness could have affected study participants’ responses differently. That being said, it is notable that in Uganda, more education does not appear to have reduced the stigma attached to HIV [110] and that the evidence for efficacy of education-based interventions in reducing mental illness stigma is also mixed [111].

The results from this study have several important applications for treatment and policy in Uganda. Primarily, it is clear that mental illness remains highly stigmatized in Uganda. Given the well-established connections between stigma and undertreatment, underfunding, and even abuse of people with mental illnesses, there remains important work to be done in reducing stigma to improve the health and lives of persons with mental illness. Second, this experiment indicates that portrayals of successful treatment of mental illness did not reduce stigmatizing attitudes in Uganda the way it seemed to reduce stigmatizing attitudes in the US. Further research, particularly qualitative investigation into the stigma attached to mental illness, is needed to achieve deeper understanding of the stigma pathways associated with mental illness throughout East Africa.

In summary, we found that mental illness stigma is common in rural southwestern Uganda. Stigma erodes efforts to promote mental health, preventing people from seeking treatment and putting them at risk for further suffering [91]. Describing mental illness as treatable does not seem to have had any effect on reducing negative attitudes toward mental illness or persons with mental illness in rural southwestern Uganda. Instead, further research into stigma reduction is necessary to understand other ways to address the stigma of mental illness in East Africa.

## Supporting information

S1 Text Vignettes presented to participants. (DOCX)

S2 Text Prespecified analysis plan for measuring beliefs and norms about persons with mental illness. (DOCX)

S3 Text Description of technical error. (DOCX)

S1 CONSORT checklist Checklist of information to include when reporting a randomized trial. (DOCX)

## Abbreviations

AOR: adjusted odds ratio
ARR: adjusted risk ratio
CASIC: Computer Assisted Survey Information Collection
LMICs: low- and middle-income countries
