# Supplementary material for: Portrayals of mental illness, treatment, and relapse and their effects on the stigma of mental illness: Population-based, randomized survey experiment in rural Uganda
Source: PLoS Med. 2019 Sep 20;16(9):e1002908. doi: 10.1371/journal.pmed.1002908 (PMC6754129; doi:10.1371/journal.pmed.1002908)
Supplement: S1 Text — (DOCX) [file pmed.1002908.s001.docx]

# S1 Text: Vignettes presented to participants

## Version 1: Control (and first paragraph of all vignettes 2-10)

Imagine there is a young Munyankole woman who has completed primary school. She has never taken alcohol or drugs, nor has she ever contracted any major medical illness such as HIV. She enjoys working in the family’s banana plantation and taking part in various activities in the community. Sometimes she might think too much about things and be sad, but this only happened twice and only for a short time. She has experienced the usual ups and downs of life, but managed to get through the challenges she has faced.

## Version 2: Schizophrenia (includes Version 1 text to begin)

One day, things started to change. She lost her drive to participate in her usual work and community activities and soon began spending most of the day alone in her room. Sometimes people in the village would see her walking alone, talking to herself even though no one was accompanying her. She stopped taking food prepared by others as she believed they were trying to poison her, but the husband and children all ate from the same bowl. She started telling her husband that she heard commanding her to do things or telling her that she has been bewitched, but she heard these voices even when the children were away at school and no one but the husband was at home. Occasionally she would argue and sometimes even abuse people, even her father in law. She stopped bathing regularly and frequently urinated on herself. One day she wandered off into the bush. Her husband spent 2 days trying to find her. When he found her, she said a big machine in Kampala was controlling her and made her go into the bush.

## Version 3: Schizophrenia + treatment with complete response (includes Version 1 text to begin)

One day, things started to change. She lost her drive to participate in her usual work and community activities and soon began spending most of the day alone in her room. Sometimes people in the village would see her walking alone, talking to herself even though no one was accompanying her. She stopped taking food prepared by others as she believed they were trying to poison her, but the husband and children all ate from the same bowl. She started telling her husband that she heard commanding her to do things or telling her that she has been bewitched, but she heard these voices even when the children were away at school and no one but the husband was at home. Occasionally she would argue and sometimes even abuse people, even her father in law. She stopped bathing regularly and frequently urinated on herself. One day she wandered off into the bush. Her husband spent 2 days trying to find her. When he found her, she said a big machine in Kampala was controlling her and made her go into the bush.

At that point, her family brought her to the hospital, where she stayed for 1 month receiving treatment, after which she felt well enough to leave the hospital. She returned for her follow up appointment one month later and continued to take medicine regularly. After 3 months she felt well enough to begin working in the banana plantation again.

## Version 4: Schizophrenia + treatment with relapse (includes Version 1 text to begin)

One day, things started to change. She lost her drive to participate in her usual work and community activities and soon began spending most of the day alone in her room. Sometimes people in the village would see her walking alone, talking to herself even though no one was accompanying her. She stopped taking food prepared by others as she believed they were trying to poison her, but the husband and children all ate from the same bowl. She started telling her husband that she heard commanding her to do things or telling her that she has been bewitched, but she heard these voices even when the children were away at school and no one but the husband was at home. Occasionally she would argue and sometimes even abuse people, even her father in law. She stopped bathing regularly and frequently urinated on herself. One day she wandered off into the bush. Her husband spent 2 days trying to find her. When he found her, she said a big machine in Kampala was controlling her and made her go into the bush.

At that point, her family brought her to the hospital, where she stayed for 1 month receiving treatment, after which she felt well enough to leave the hospital. She returned for her follow up appointment one month later and continued to take medicine regularly. After 3 months she felt well enough to begin working in the banana plantation again. However, several months later she and her family could no longer afford to pay for her medicine. Her illness soon returned and her family brought her back to the hospital.

## Version 5: Bipolar (includes Version 1 text to begin)

One day, she started to become very restless and irritable and often stayed awake at night when everyone else was asleep. Others found her to be over talkative, and when she talked people could not understand her because she talked too fast and talked about too many different ideas and plans. Sometimes it was almost as if she was too happy. She would speak of herself as a very important and powerful woman, telling others that she had lots of land and hundreds of cows. But this was not true, because according to the husband they were a typical Ugandan family with only 3 small plots of land and 2 cows. She began telling people that she had developed a cure for AIDS and was planning to go and tell the president. In preparation for her trip, she began trying to sell all of the family’s possessions.

## Version 6: Bipolar + treatment with complete response (includes Version 1 text to begin)

One day, she started to become very restless and irritable and often stayed awake at night when everyone else was asleep. Others found her to be over talkative, and when she talked people could not understand her because she talked too fast and talked about too many different ideas and plans. Sometimes it was almost as if she was too happy. She would speak of herself as a very important and powerful woman, telling others that she had lots of land and hundreds of cows. But this was not true, because according to the husband they were a typical Ugandan family with only 3 small plots of land and 2 cows. She began telling people that she had developed a cure for AIDS and was planning to go and tell the president. In preparation for her trip, she began trying to sell all of the family’s possessions.

At that point, her family brought her to the hospital, where she stayed for 1 month receiving treatment, after which she felt well enough to leave the hospital. She returned for her follow up appointment one month later and continued to take medicine regularly. After 3 months she felt well enough to begin working in the banana plantation again.

## Version 7: Bipolar + treatment with relapse (includes Version 1 text to begin)

One day, she started to become very restless and irritable and often stayed awake at night when everyone else was asleep. Others found her to be over talkative, and when she talked people could not understand her because she talked too fast and talked about too many different ideas and plans. Sometimes it was almost as if she was too happy. She would speak of herself as a very important and powerful woman, telling others that she had lots of land and hundreds of cows. But this was not true, because according to the husband they were a typical Ugandan family with only 3 small plots of land and 2 cows. She began telling people that she had developed a cure for AIDS and was planning to go and tell the president. In preparation for her trip, she began trying to sell all of the family’s possessions.

At that point, her family brought her to the hospital, where she stayed for 1 month receiving treatment, after which she felt well enough to leave the hospital. She returned for her follow up appointment one month later and continued to take medicine regularly. After 3 months she felt well enough to begin working in the banana plantation again. However, several months later she and her family could no longer afford to pay for her medicine. Her illness soon returned and her family brought her back to the hospital.

## Version 8: Depression (includes Version 1 text to begin)

One day, she started to feel very sad. She began to wake up in the morning with a flat, heavy feeling that remained with her all day long. She lost her drive to participate in her usual work and community activities and soon began spending most of the day alone in her room. In fact, nothing seemed to give her pleasure. Even when good things happened, like when the family had a good harvest, she could not feel happy. She began to find it extremely difficult to accomplish anything, and it seemed like life was such a burden. She lacked energy all the time and felt tired, but when night came she found it a struggle to sleep. She found that she could not concentrate on things, and felt like she was thinking too much. She felt like her life was worthless. She even thought about taking her own life.

## Version 9: Depression + treatment with complete response (includes Version 1 text to begin)

One day, she started to feel very sad. She began to wake up in the morning with a flat, heavy feeling that remained with her all day long. She lost her drive to participate in her usual work and community activities and soon began spending most of the day alone in her room. In fact, nothing seemed to give her pleasure. Even when good things happened, like when the family had a good harvest, she could not feel happy. She began to find it extremely difficult to accomplish anything, and it seemed like life was such a burden. She lacked energy all the time and felt tired, but when night came she found it a struggle to sleep. She found that she could not concentrate on things, and felt like she was thinking too much. She felt like her life was worthless. She even thought about taking her own life.

At that point, her family brought her to the hospital, where she stayed for 1 month receiving treatment, after which she felt well enough to leave the hospital. She returned for her follow up appointment one month later and continued to take medicine regularly. After 3 months she felt well enough to begin working in the banana plantation again.

## Version 10: Depression + treatment with relapse (includes Version 1 text to begin)

One day, she started to feel very sad. She began to wake up in the morning with a flat, heavy feeling that remained with her all day long. She lost her drive to participate in her usual work and community activities and soon began spending most of the day alone in her room. In fact, nothing seemed to give her pleasure. Even when good things happened, like when the family had a good harvest, she could not feel happy. She began to find it extremely difficult to accomplish anything, and it seemed like life was such a burden. She lacked energy all the time and felt tired, but when night came she found it a struggle to sleep. She found that she could not concentrate on things, and felt like she was thinking too much. She felt like her life was worthless. She even thought about taking her own life.

At that point, her family brought her to the hospital, where she stayed for 1 month receiving treatment, after which she felt well enough to leave the hospital. She returned for her follow up appointment one month later and continued to take medicine regularly. After 3 months she felt well enough to begin working in the banana plantation again. However, several months later she and her family could no longer afford to pay for her medicine. Her illness soon returned and her family brought her back to the hospital.
