# Supplementary material for: Portrayals of mental illness, treatment, and relapse and their effects on the stigma of mental illness: Population-based, randomized survey experiment in rural Uganda
Source: PLoS Med. 2019 Sep 20;16(9):e1002908. doi: 10.1371/journal.pmed.1002908 (PMC6754129; doi:10.1371/journal.pmed.1002908)
Supplement: S2 Text — (DOCX) [file pmed.1002908.s002.docx]

S2 Text: Prespecified Analysis Plan for Measuring Beliefs and Norms About Persons with Mental Illness

ClinicalTrials.gov Identifier: NCT03656770

November 2016

* Note: The experimental procedures for this study were registered with ClinicalTrials.gov. The protocol record was entered in April 2017 but, due to an administrative error, was not released until August 2018.

# Inclusion Criteria

Eligibility to participate in this randomized survey experiment will be open to all adults aged 18 years and above (and emancipated minors aged 16-17 years) who maintain stable primary residence within the roughly 11 square kilometer area of Nyakabare Parish, Mbarara, Uganda, who can give informed consent to participate, and who participated in the 2014-15 baseline survey.

# Exclusion Criteria

Exclusions include people who cannot communicate meaningfully with research staff, e.g., due to deafness, mutism, or aphasia; people with behavioral problems thought to represent psychosis, neurological damage, or acute intoxication; and people too cognitively impaired to provide informed consent.

# Intervention

Study participants will be randomly assigned to receive one of 10 different vignettes describing a young woman. Treatment assignment in a parallel group design following a 1:1:1:1:1:1:1:1:1:1 allocation ratio will be determined centrally using a computerized random number generator. Neither the research assistants administering the questionnaires nor the study participants will be aware of the vignettes to which the study participants are assigned. The research assistants will not be blinded, however, so it is possible that they may perceive the differences in the vignettes administered to different study participants. To ensure balance across sex and village strata, we will generate 16 separate randomization schedules for subsets of participants defined by strata of sex and village of residence.

Each version of the questionnaire portrays a young Ugandan woman with different profiles of illness severity, treatment, and treatment response. The control vignette will describe the demographic characteristics and basic life story of a typical Ugandan woman with no further elaboration. The remaining nine vignettes will include the same basic description of the woman but also describe her experiencing three different types of symptoms (psychosis, mania, and depression, suggestive of schizophrenia, bipolar disorder, and major depressive disorder, respectively), each with three different treatment outcomes (no treatment, successful treatment followed by recovery, and successful treatment followed by recovery and then relapse/recurrence). These vignettes were adapted from McGinty and colleagues to fit the local context based on feedback from key informants, documented symptom presentation in Uganda, and consultation with psychiatrists at the Mbarara Regional Referral Hospital.

The treatment assignment arms will be as follows:

1. No Intervention: V1: Control
   - This version of the survey questionnaire will depict a young woman with no symptoms of mental illness.
2. Experimental: V2: Schizophrenia
   - This version of the survey questionnaire will depict a young woman with untreated and symptomatic schizophrenia.
3. Experimental: V3: Schizophrenia + Treatment with Response
   - This version of the survey questionnaire will depict a young woman with schizophrenia, successfully treated with complete response.
4. Experimental: V4: Schizophrenia + Treatment with Response, Followed by Relapse
   - This version of the survey questionnaire will depict a young woman with schizophrenia, successfully treated with partial relapse.
5. Experimental: Version 5: Bipolar
   - This version of the survey questionnaire will depict a young woman with untreated and symptomatic bipolar disorder.
6. Experimental: V6: Bipolar + Treatment with Response
   - This version of the survey questionnaire will depict a young woman with bipolar disorder, successfully treated with complete response.
7. Experimental: V7: Bipolar + Treatment with Response, Followed by Relapse
   - This version of the survey questionnaire will depict a young woman with bipolar disorder, successfully treated with partial relapse.
8. Experimental: V8: Depression
   - This version of the survey questionnaire will depict a young woman with untreated and symptomatic major depressive disorder.
9. Experimental: V9: Depression + Treatment with Response
   - This version of the survey questionnaire will depict a young woman with major depressive disorder, successfully treated with complete response.
10. Experimental: V10: Depression + Treatment with Response, Followed by Relapse
    - This version of the survey questionnaire will depict a young woman with major depressive disorder, successfully treated with partial relapse.

# Outcome Variables

After being presented with the experimental vignettes, study participants will be asked three questions regarding their personal beliefs about mental illness and about people with mental illness, and three questions regarding their perceptions of village norms about mental illness and about people with mental illness. Each of these outcome variables is a single-item, culturally-adapted instrument developed specifically for this study.

1. Social Distance: Willingness to have the woman portrayed in the vignette to marry into the study participant's family
2. Perceived Norms about Social Distance: Study participant’s perception of the extent to which other people would be willing to have the woman portrayed in the vignette to marry into their families
3. Negative Attitudes (Attribution): Personal belief that the symptoms of the woman portrayed in the vignette represent divine punishment
4. Perceived Norms about Negative Attitudes (Attribution): Study participant’s perception of the extent to which other people believe that the symptoms of the woman portrayed in the vignette represent divine punishment
5. Negative Attitudes (Shame): Personal belief that the symptoms of the woman portrayed in the vignette bring shame upon her family
6. Perceived Norms about Negative Attitudes (Shame): Study participant’s perception of the extent to which other people believe that the symptoms of the woman portrayed in the vignette bring shame upon her family

In response to the three items about personal beliefs, participants will be permitted to provide one of five different responses: “Yes”, “No”, “It depends on knowing more details”, “Do not know”, and “Refuse to respond”. In response to the three items about perceived norms, participants will be permitted to provide responses on a four-point Likert-type scale (in addition to “Do not know” and “Refuse to respond”): “All or almost all”, “More than half”, “Fewer than half”, and “Very few, or no one”.

# Primary Analysis Plan

For the three outcome variables measuring personal beliefs, responses will be coded such that 0 denotes a non-stigmatizing response and 1 denotes a stigmatizing or ambivalent response. Thus, unwillingness to allow the woman to marry a member of the participant’s family, belief that she is receiving divine punishment, and belief that she brings shame on her family will be assigned values of 1, along with the ambivalent response of “it depends.” Correspondingly, willingness to allow the woman to marry into the family, belief that she is not receiving divine punishment, and belief that she does not bring shame upon her family will be assigned values of 0. “Do not know” and “refuse” will be considered missing data. We will fit Poisson regression models specifying each outcome as the dependent variable and the vignette treatment assignment as the primary exposure of interest. The regression models will additionally include adjustment for sex and village of residence to ensure accurate confidence intervals that account for the stratified randomization scheme. Cluster-correlated robust estimates of variance will be used so that the estimated incidence rate ratios can be interpreted as risk ratios.

For the three 4-level categorical outcome variables measuring perceived norms, responses will coded 1-4 such that the lowest category indicates the least perceived stigma and the highest category indicates the most perceived stigma. (For example, in response to the question about whether others in the village would permit the woman in the vignette to marry into the family, “Very few, or no one” will be designated as the highest category and “All or almost all” will be designated as the lowest category.) “Do not know” and “refuse” will be considered missing data. We will fit ordinal logit regression models specifying each outcome as the dependent variable and the vignette treatment assignment as the primary exposure of interest. The regression models will additionally include adjustment for sex and village of residence to ensure accurate confidence intervals that account for the stratified randomization scheme. The exponentiated regression coefficients will be interpreted as estimated odds ratios. To confirm that the regression coefficients do not vary across the logit equations, we will the omnibus Wald test by Brant. Should the assumption of proportional odds fail to hold, we will explore other regression models as appropriate (e.g., generalized ordered logistic regression).

# Subgroup Analyses

No subgroup analyses are planned.

# Sensitivity Analyses

No sensitivity analyses are planned.

* During the peer review process, several reviewers raised comments about the prespecified analysis plan that required us to conduct sensitivity analyses to probe the robustness of our findings. These are described in more detail below.

## Sensitivity Analysis Conducted in Response to Reviewer Comments: Combining Treatment and Relapse Arms (May 2019)

In response to a comment raised by a reviewer on the initial submission, we reanalyzed our data grouping the two treatment arms together: instead of being analyzed as separate treatment arms, the “mental illness + treatment” arm is grouped together with the “mental illness + treatment + relapse” arm. The results of these analyses, presented in **Tables A-D** below, demonstrate that the results do not differ substantially when the data are grouped in this way. (Tables A-D correspond to manuscript Tables 2-5.)

**Table A. Stigmatizing personal beliefs, by treatment assignment (with “treatment” and “treatment + relapse” grouped together).**

*This table presents findings that are analogous to the results presented in manuscript Table 2.*

| **Stigmatizing personal beliefs** | | **Unwilling for family member to marry ^a^** | **Is receiving divine punishment ^a^** | **Brings shame on family ^a^** |
| --- | --- | --- | --- | --- |
| Control | | 37 (30%) | 27 (22%) | 29 (23%) |
| Schizophrenia | Mental Illness | 120 (86%) | 58 (41%) | 90 (64%) |
| + Treatment/Relapse | | 369 (84%) | 164 (37%) | 242 (55%) |
| Bipolar | Mental Illness | 116 (87%) | 72 (54%) | 98 (73%) |
| + Treatment/Relapse | | 169 (79%) | 79 (37%) | 117 (55%) |
| Depression | Mental Illness | 82 (80%) | 36 (35%) | 62 (61%) |
| + Treatment/Relapse | | 166 (83%) | 70 (35%) | 105 (53%) |

^a^ N (%) refer to the number and proportion of study participants assigned to each treatment arm who endorsed the stigmatizing belief shown in the column header. Column percentages do not add to 100% because each column represents a different outcome variable (i.e., the columns do not represent categories of a single categorical variable).

**Table B. Perceived stigmatizing beliefs of others, by treatment assignment (with “treatment” and “treatment + relapse” grouped together).**

*This table presents findings that are analogous to the results presented in manuscript Table 3.*

| **Perception that most others (>50% of others) hold stigmatizing belief ^a^** | | **Most others unwilling for family member to marry ^b^** | **Most others believe receiving divine punishment ^b^** | **Most others believe Brings shame on family ^b^** |
| --- | --- | --- | --- | --- |
| Control | | 38 (30%) | 25 (20%) | 23 (18%) |
| Schizophrenia | Mental Illness | 114 (81%) | 39 (28%) | 59 (42%) |
| + Treatment/Relapse | | 370 (84%) | 126 (29%) | 188 (43%) |
| Bipolar | Mental Illness | 117 (87%) | 50 (37%) | 67 (50%) |
| + Treatment/Relapse | | 179 (84%) | 52 (24%) | 88 (41%) |
| Depression | Mental Illness | 76 (75%) | 32 (31%) | 41 (40%) |
| + Treatment/Relapse | | 169 (85%) | 54 (27%) | 78 (39%) |

^a^ The numbers and percentages in each cell refer to the percentage of study participants who believe that most others (>50% of others) in their village hold the stigmatizing belief in question

^b^ N (%) refer to the number and proportion of study participants assigned to each treatment arm who endorsed the stigmatizing belief shown in the column header. Column percentages do not add to 100% because each column represents a different outcome variable (i.e., the columns do not represent categories of a single categorical variable)

**Table C. Risk of stigmatizing personal beliefs, by treatment assignment, based on Poisson regression (with “treatment” and “treatment + relapse” grouped together).**

*This table presents findings that are analogous to the results presented in manuscript Table 4.*

| **Stigmatizing personal beliefs** | | **Unwilling for family member to marry** | | **Is receiving divine punishment** | | **Brings shame on family** | |
| --- | --- | --- | --- | --- | --- | --- | --- |
|  |  | **ARR (95% CI)** | ***p*-value** | **ARR (95% CI)** | ***p*-value** | **ARR (95% CI)** | ***p*-value** |
| Control |  | Ref. |  | Ref. |  | Ref. |  |
| Schizophrenia | Mental Illness | 2.9 (2.0-4.2) | <0.001 | 2.0 (1.3-2.9) | 0.001 | 2.8 (2.0-3.8) | <0.001 |
| + Treatment/Relapse | | 2.8 (1.9-4.3) | <0.001 | 1.8 (1.1-2.8) | 0.023 | 2.4 (1.6-3.5) | <0.001 |
| Bipolar | Mental Illness | 3.0 (1.9-4.5) | <0.001 | 2.5 (1.9-3.3) | <0.001 | 3.1 (2.2-4.4) | <0.001 |
| + Treatment/Relapse | | 2.7 (1.9-3.8) | <0.001 | 1.7 (1.2-2.4) | 0.002 | 2.4 (1.7-3.2) | <0.001 |
| Depression | Mental Illness | 2.7 (1.8-4.1) | <0.001 | 1.7 (1.2-2.3) | 0.002 | 2.6 (1.8-3.7) | <0.001 |
| + Treatment/Relapse | | 2.8 (1.9-4.3) | <0.001 | 1.6 (1.0-2.7) | 0.051 | 2.3 (1.6-3.3) | <0.001 |

**Table D. Odds of perceiving stigmatizing beliefs of others, by treatment assignment, based on ordered logit regression (with “treatment” and “treatment + relapse” grouped together).**

*This table presents findings that are analogous to the results presented in manuscript Table 5.*

| **Perceived norms** | | **Most others unwilling for family member to marry** | | **Most others believe she is receiving divine punishment** | | **Most others believe she brings shame on family** | |
| --- | --- | --- | --- | --- | --- | --- | --- |
|  |  | **AOR (95% CI)*** | ***p-value*** | **AOR (95% CI)** | ***p-value*** | **AOR (95% CI)** | ***p-value*** |
| Control | Ref. |  |  |  |  |  |  |
| Schizophrenia | Mental Illness | 14.0 (6.2-31.3) | <0.001 | 2.1 (1.5-2.9) | <0.001 | 3.8 (2.0-7.1) | <0.001 |
| + Treatment/Relapse | | 13.0 (6.1-27.7) | <0.001 | 2.2 (1.5-3.1) | <0.001 | 3.7 (2.3-5.8) | <0.001 |
| Bipolar | Mental Illness | 16.2 (7.0-37.7) | <0.001 | 3.1 (2.1-4.6) | <0.001 | 5.1 (4.0-6.4) | <0.001 |
| + Treatment/Relapse | | 9.8 (4.5-21.7) | <0.001 | 1.9 (1.3-2.7) | <0.001 | 3.3 (2.2-4.7) | <0.001 |
| Depression | Mental Illness | 6.9 (2.5-18.9) | <0.001 | 2.8 (1.8-4.3) | <0.001 | 3.3 (1.9-5.9) | <0.001 |
| + Treatment/Relapse | | 11.3 (5.4-23.6) | <0.001 | 2.1 (1.5-2.9) | <0.001 | 3.3 (2.0-5.4) | <0.001 |

* The estimated adjusted odds ratios in each cell refer to the relative odds of being in a higher category of perceiving that more people in their village (ranging from “very few, or no one” to “all or almost all”) hold the stigmatizing belief in question.

## Sensitivity Analysis Conducted in Response to Reviewer Comments: Coding Ambivalent Responses as Missing or as Non-Stigmatizing (May 2019)

On the initial submission, two reviewers raised a concern about how ambivalent responses were coded. The number of ambivalent responses was quite small as can be observed in the disaggregated responses in **Tables E-I** below.

**Table E. Stigmatizing personal beliefs (“unwilling for family member to marry” only), by treatment assignment, including ambivalent and missing responses.**

*This table presents findings that are analogous to the results presented in column 1 of manuscript Table 2. In manuscript Table 2, the “Yes” responses are grouped with the ambivalent response “It depends”. In the table below, the ambivalent responses are shown separately, as are the “don’t know” and “refused” responses.*

| **Stigmatizing personal beliefs** | | **Yes** | **No** | **Depends** | **Don’t Know** | **Refused** |
| --- | --- | --- | --- | --- | --- | --- |
| Control | | 32 (26%) | 88 (70%) | 5 (4%) | 0 (0%) | 0 (0%) |
| Schizophrenia | Mental Illness | 117 (84%) | 20 (14%) | 3 (2%) | 0 (0%) | 0 (0%) |
|  | + Treatment | 116 (83%) | 20 (14%) | 4 (3%) | 0 (0%) | 0 (0%) |
|  | + Relapse | 240 (80%) | 50 (17%) | 9 (3%) | 0 (0%) | 1 (<1%) |
| Bipolar | Mental Illness | 112 (84%) | 17 (13%) | 4 (3%) | 1 (1%) | 0 (0%) |
|  | + Treatment | 96 (77%) | 27 (22%) | 2 (2%) | 0 (0%) | 0 (0%) |
|  | + Relapse | 66 (74%) | 18 (20%) | 5 (6%) | 0 (0%) | 0 (0%) |
| Depression | Mental Illness | 79 (77%) | 20 (20%) | 3 (3%) | 0 (0%) | 0 (0%) |
|  | + Treatment | 78 (76%) | 21 (20%) | 2 (2%) | 1 (1%) | 0 (0%) |
|  | + Relapse | 83 (85%) | 12 (12%) | 3 (3%) | 0 (0%) | 0 (0%) |

**Table F. Stigmatizing personal beliefs (“is receiving divine punishment” only), by treatment assignment, including ambivalent and missing responses.**

*This table presents findings that are analogous to the results presented in column 2 of manuscript Table 2. In manuscript Table 2, the “Yes” responses are grouped with the ambivalent response “It depends”. In the table below, the ambivalent responses are shown separately, as are the “don’t know” and “refused” responses.*

| **Stigmatizing personal beliefs** | | **Yes** | **No** | **Depends** | **Don’t Know** | **Refuses** |
| --- | --- | --- | --- | --- | --- | --- |
| Control | | 27 (22%) | 93 (74%) | 4 (3%) | 1 (1%) | 0 (0%) |
| Schizophrenia | Mental Illness | 58 (41%) | 69 (49%) | 8 (6%) | 5 (4%) | 0 (0%) |
|  | +Treatment | 56 (40%) | 73 (52%) | 9 (6%) | 2 (1%) | 0 (0%) |
|  | + Relapse | 108 (36%) | 170 (57%) | 11 (4%) | 10 (3%) | 1 (0%) |
| Bipolar | Mental Illness | 72 (54%) | 53 (40%) | 8 (6%) | 1 (1%) | 0 (0%) |
|  | +Treatment | 50 (40%) | 70 (56%) | 3 (2%) | 2 (2%) | 0 (0%) |
|  | + Relapse | 29 (33%) | 53 (60%) | 5 (6%) | 2 (2%) | 0 (0%) |
| Depression | Mental Illness | 36 (35%) | 56 (55%) | 8 (8%) | 2 (2%) | 0 (0%) |
|  | +Treatment | 40 (39%) | 58 (57%) | 2 (2%) | 2 (2%) | 0 (0%) |
|  | + Relapse | 30 (31%) | 58 (59%) | 7 (7%) | 3 (3%) | 0 (0%) |

**Table G. Stigmatizing personal beliefs (“brings shame upon family” only), by treatment assignment, including ambivalent and missing responses.**

*This table presents findings that are analogous to the results presented in column 3 of manuscript Table 2. In manuscript Table 2, the “Yes” responses are grouped with the ambivalent response “It depends”. In the table below, the ambivalent responses are shown separately, as are the “don’t know” and “refused” responses.*

| **Stigmatizing personal beliefs** | | **Yes** | **No** | **Depends** | **Don’t Know** | **Refuses** |
| --- | --- | --- | --- | --- | --- | --- |
| Control | | 29 (23%) | 93 (74%) | 3 (2%) | 0 (0%) | 0 (0%) |
| Schizophrenia | Mental Illness | 90 (64%) | 47 (34%) | 2 (1%) | 1 (1%) | 0 (0%) |
|  | +Treatment | 72 (51%) | 65 (46%) | 3 (2%) | 0 (0%) | 0 (0%) |
|  | + Relapse | 170 (57%) | 126 (42%) | 1 (<1%) | 2 (1%) | 1 (0%) |
| Bipolar | Mental llness | 98 (73%) | 31 (23%) | 5 (4%) | 0 (0%) | 0 (0%) |
|  | +Treatment | 68 (54%) | 55 (44%) | 2 (2%) | 0 (0%) | 0 (0%) |
|  | + Relapse | 49 (55%) | 38 (43%) | 1 (1%) | 1 (1%) | 0 (0%) |
| Depression | Mental llness | 62 (61%) | 38 (37%) | 2 (2%) | 0 (0%) | 0 (0%) |
|  | +Treatment | 55 (54%) | 45 (44%) | 1 (1%) | 1 (1%) | 0 (0%) |
|  | + Relapse | 50 (51%) | 46 (47%) | 2 (2%) | 0 (0%) | 0 (0%) |

In response to the reviewers’ comments, we included two sensitivity analyses with ambivalent responses coded as missing (Table H) and as non-stigmatizing (Table I) to demonstrate that the primary findings presented in manuscript Table 4 are insensitive to how the ambivalent responses are coded.

**Table H. Risk of stigmatizing personal beliefs, by treatment assignment, based on Poisson regression (with “Depends” coded as missing).**

*This table presents findings that are analogous to the results presented in manuscript Table 4.*

| **Stigmatizing personal beliefs** | | **Unwilling for family member to marry** | | **Is receiving divine punishment** | | **Brings shame on family** | |
| --- | --- | --- | --- | --- | --- | --- | --- |
|  |  | **ARR (95% CI)** | ***p*-value** | **ARR (95% CI)** | ***p*-value** | **ARR (95% CI)** | ***p*-value** |
| Control |  | Ref. |  | Ref. |  | Ref. |  |
| Schizophrenia | Mental Illness | 3.2 (2.0-5.0) | <0.001 | 2.0 (1.3-3.0) | 0.001 | 2.8 (2.0-3.8) | <0.001 |
|  | + Treatment | 3.2 (1.9-5.4) | <0.001 | 1.9 (1.1-3.4) | 0.024 | 2.2 (1.4-3.5) | 0.001 |
|  | + Relapse | 3.1 (1.9-5.0) | <0.001 | 1.7 (1.1-2.7) | 0.019 | 2.4 (1.7-3.4) | <0.001 |
| Bipolar | Mental Illness | 3.3 (2.0-5.4) | <0.001 | 2.6 (1.9-3.4) | <0.001 | 3.2 (2.3-4.5) | <0.001 |
|  | + Treatment | 2.9 (2.0-4.4) | <0.001 | 1.8 (1.3-2.5) | <0.001 | 2.3 (1.7-3.3) | <0.001 |
|  | + Relapse | 3.0 (1.8-4.9) | <0.001 | 1.6 (1.1-2.3) | 0.019 | 2.4 (1.8-3.2) | <0.001 |
| Depression | Mental Illness | 2.7 (1.8-4.9) | <0.001 | 1.7 (1.3-2.3) | <0.001 | 2.6 (1.8-3.7) | <0.001 |
|  | + Treatment | 2.7 (1.8-4.9) | <0.001 | 1.8 (1.1-3.1) | 0.028 | 2.3 (1.5-3.5) | <0.001 |
|  | + Relapse | 3.0 (2.0-5.4) | <0.001 | 1.5 (0.9-2.7) | 0.137 | 2.2 (1.5-3.3) | <0.001 |

**Table I. Risk of stigmatizing personal beliefs, by treatment assignment, based on Poisson regression (with “Depends” coded as non-stigmatizing).**

| **Stigmatizing personal beliefs** | | **Unwilling for family member to marry** | | **Is receiving divine punishment** | | **Brings shame on family** | |
| --- | --- | --- | --- | --- | --- | --- | --- |
|  |  | **ARR (95% CI)** | ***p*-value** | **ARR (95% CI)** | ***p*-value** | **ARR (95% CI)** | ***p*-value** |
| Control |  | Ref. |  | Ref. |  | Ref. |  |
| Schizophrenia | Mental Illness | 3.3 (2.0-5.2) | <0.001 | 1.9 (1.3-2.9) | 0.001 | 2.6 (1.9-3.5) | <0.001 |
|  | + Treatment | 3.2 (1.9-5.6) | <0.001 | 1.9 (1.1-3.1) | 0.015 | 2.1 (1.4-3.1) | <0.001 |
|  | + Relapse | 3.1 (1.9-5.2) | <0.001 | 1.6 (1.1-2.5) | 0.025 | 2.3 (1.7-3.0) | <0.001 |
| Bipolar | Mental Illness | 3.3 (2.0-5.5) | <0.001 | 2.4 (1.8-3.3) | <0.001 | 3.0 (2.2-4.0) | <0.001 |
|  | + Treatment | 3.0 (2.0-4.6) | <0.001 | 1.7 (1.3-2.3) | <0.001 | 2.2 (1.6-3.0) | <0.001 |
|  | + Relapse | 2.9 (1.7-4.8) | <0.001 | 1.6 (1.1-2.2) | 0.007 | 2.2 (1.8-2.8) | <0.001 |
| Depression | Mental Illness | 3.0 (1.8-5.2) | <0.001 | 1.8 (1.4-2.2) | <0.001 | 2.4 (1.7-3.4) | <0.001 |
|  | + Treatment | 3.0 (1.8-5.0) | <0.001 | 1.7 (1.0-2.7) | 0.036 | 2.2 (1.5-3.2) | <0.001 |
|  | + Relapse | 3.3 (2.1-5.4) | <0.001 | 1.6 (1.0-2.5) | 0.07 | 2.1 (1.4-3.1) | <0.001 |
