# Supplementary material for: Portrayals of mental illness, treatment, and relapse and their effects on the stigma of mental illness: Population-based, randomized survey experiment in rural Uganda
Source: PLoS Med. 2019 Sep 20;16(9):e1002908. doi: 10.1371/journal.pmed.1002908 (PMC6754129; doi:10.1371/journal.pmed.1002908)
Supplement: S3 Text — (DOCX) [file pmed.1002908.s003.docx]

# S3 Text: Description of technical error

A technical error occurred after the vignette versions had been randomly assigned (**Table A**). The result of the error was that 1 participant assigned to version 2, 58 participants assigned to version 7, 60 assigned to version 8, 63 assigned to version 9, and 69 assigned to version 10 were erroneously re-assigned to instead receive version 4. One participant assigned to version 1 was erroneously re-assigned to version 2, and 9 participants assigned to receive version 1 was erroneously re-assigned to version 4. Altogether, this technical error resulted in a greater number of participants receiving version 4 than any other group and substantially fewer participants receiving versions 7, 8, 9, and 10.

**Table A. Tabular description of assignment error.**

|  |  | Intended random assignment | | | | | | | | | | **Actual** | |  |
| --- | --- | --- | --- | --- | --- | --- | --- | --- | --- | --- | --- | --- | --- | --- |
|  |  | **1** | **2** | **3** | **4** | **5** | **6** | **7** | **8** | **9** | **10** | Pool | Interviewed | |
| Actual assignment | **1** | *171* |  |  |  |  |  |  |  |  |  | 171 | 134* | |
|  | **2** | *1* | *179* |  |  |  |  |  |  |  |  | 180 | 140 | |
|  | **3** |  |  | 176 |  |  |  |  |  |  |  | 176 | 140 | |
|  | **4** |  | *1* |  | 178 |  |  | ***58*** | ***60*** | ***63*** | ***69*** | ***429*** | **291** | |
|  | **5** |  |  |  |  | 177 |  |  |  |  |  | 177 | 134 | |
|  | **6** |  |  |  |  |  | 175 |  |  |  |  | 175 | 125 | |
|  | **7** |  |  |  |  |  |  | ***116*** |  |  |  | ***116*** | **89** | |
|  | **8** |  |  |  |  |  |  |  | ***121*** |  |  | ***121*** | **102** | |
|  | **9** |  |  |  |  |  |  |  |  | ***115*** |  | ***115*** | **102** | |
|  | **10** |  |  |  |  |  |  |  |  |  | ***116*** | ***116*** | **98** | |
| **Intended** | Pool | 172 | 180 | 176 | 178 | 177 | 175 | 174 | 181 | 178 | 185 | **1776** |  | |
|  | Interviewed | 126^a^ | 139 | 140 | 150 | 134 | 125 | 121 | 138 | 139 | 143 |  | **1355** | |

^a^ During interviews, 9 participants assigned to version 1 were presented with version 4 instead.

In-depth investigation of the technical error revealed that the re-assignment did not follow a systematic process (i.e., the error was non-differential). Demographics of the correctly and incorrectly assigned groups are compared in **Table B**. Compared with study participants who had been correctly assigned, those who had been incorrectly assigned differed slightly in their sex, marital status, age, and geographical distributions. A more significant difference in their educational profiles was noted.

**Table B. Demographic comparison of correctly and incorrectly assigned participants.**

|  | **Correctly assigned** | | **Incorrectly assigned** | |
| --- | --- | --- | --- | --- |
|  | N | % | N | % |
| **Sex** |  |  |  |  |
| Male | 537 | 45% | 62 | 41% |
| Female | 667 | 55% | 89 | 59% |
| **Education** |  |  |  |  |
| None | 190 | 16% | 7 | 4% |
| Some primary (P1-P6) | 360 | 30% | 33 | 22% |
| Completed primary (P7) | 296 | 24% | 36 | 24% |
| Beyond primary (S1-S6, vocational, university) | 358 | 30% | 76 | 50% |
| **Marital status** |  |  |  |  |
| Single | 425 | 35% | 59 | 39% |
| Married/cohabiting | 779 | 65% | 92 | 61% |
| **Age category** |  |  |  |  |
| 18-25 years | 164 | 14% | 45 | 30% |
| 26-35 years | 288 | 24% | 49 | 32% |
| 36-45 years | 244 | 20% | 30 | 20% |
| 46-55 years | 231 | 19% | 17 | 11% |
| 56+ years | 253 | 21% | 10 | 7% |
| Unknown | 24 | 2% | 0 | 0% |
| **Village** |  |  |  |  |
| 1 | 193 | 16% | 17 | 11% |
| 2 | 173 | 14% | 19 | 13% |
| 3 | 161 | 13% | 16 | 11% |
| 4 | 132 | 11% | 24 | 16% |
| 5 | 103 | 9% | 7 | 5% |
| 6 | 166 | 14% | 36 | 24% |
| 7 | 105 | 9% | 7 | 5% |
| 8 | 171 | 14% | 25 | 17% |

We conducted a sensitivity analysis by excluding the 151 incorrectly assigned participants and retabulating the outcomes and refitting the regression models to the data (**Tables C, D, E, and F**). Any differences were not substantive and changed neither the reported results nor the final conclusions.

**Table C. Stigmatizing personal beliefs, by treatment assignment (excluding 151 incorrectly reassigned participants).**

| **Stigmatizing personal beliefs** | | **Unwilling for family member to marry ^a^** | **Is receiving divine punishment ^a^** | **Brings shame on family ^a^** |
| --- | --- | --- | --- | --- |
| Control | | 37 (30%) | 27 (22%) | 29 (23%) |
| Schizophrenia | Mental Illness | 119 (86%) | 57 (41%) | 89 (64%) |
|  | + Treatment | 120 (86%) | 56 (40%) | 72 (51%) |
|  | + Relapse | 128 (86%) | 62 (41%) | 90 (60%) |
| Bipolar | Mental Illness | 116 (87%) | 72 (54%) | 98 (73%) |
|  | + Treatment | 98 (78%) | 50 (40%) | 68 (54%) |
|  | + Relapse | 71 (80%) | 29 (33%) | 49 (55%) |
| Depression | Mental Illness | 82 (80%) | 36 (35%) | 62 (61%) |
|  | + Treatment | 80 (78%) | 40 (39%) | 55 (54%) |
|  | + Relapse | 86 (88%) | 30 (31%) | 50 (51%) |

*^a^ N (%) refer to the number and proportion of study participants assigned to each treatment arm who endorsed the stigmatizing belief shown in the column header. Column percentages do not add to 100% because each column represents a different outcome variable (i.e., the columns do not represent categories of a single categorical variable).*

**Table D. Perceived stigmatizing beliefs of others, by treatment assignment (excluding 151 incorrectly reassigned participants).**

| **Perception that most others (>50% of others) hold stigmatizing belief ^a^** | | **Most others unwilling for family member to marry ^b^** | **Most others believe receiving divine punishment ^b^** | **Most others believe Brings shame on family ^b^** |
| --- | --- | --- | --- | --- |
| Control | | 38 (30%) | 25 (20%) | 23 (18%) |
| Schizophrenia | Mental Illness | 114 (82%) | 38 (27%) | 58 (42%) |
|  | + Treatment | 113 (81%) | 47 (34%) | 62 (44%) |
|  | + Relapse | 132 (88%) | 50 (33%) | 66 (44%) |
| Bipolar | Mental Illness | 117 (87%) | 50 (37%) | 67 (50%) |
|  | + Treatment | 104 (83%) | 31 (25%) | 51 (41%) |
|  | + Relapse | 75 (84%) | 21 (24%) | 37 (42%) |
| Depression | Mental Illness | 76 (75%) | 32 (31%) | 41 (40%) |
|  | + Treatment | 86 (84%) | 33 (32%) | 36 (35%) |
|  | + Relapse | 83 (85%) | 21 (21%) | 42 (43%) |

*^a^ The numbers and percentages in each cell refer to the percentage of study participants who believe that most others (>50% of others) in their village hold the stigmatizing belief in question*

*^b^ N (%) refer to the number and proportion of study participants assigned to each treatment arm who endorsed the stigmatizing belief shown in the column header. Column percentages do not add to 100% because each column represents a different outcome variable (i.e., the columns do not represent categories of a single categorical variable)*

**Table E. Risk of stigmatizing personal beliefs, by treatment assignment, based on Poisson regression (excluding 151 incorrectly reassigned participants).**

| **Stigmatizing personal beliefs** | | **Unwilling for family member to marry** | | **Is receiving divine punishment** | | **Brings shame on family** | |
| --- | --- | --- | --- | --- | --- | --- | --- |
|  |  | **ARR (95% CI)** | ***p*-value** | **ARR (95% CI)** | ***p*-value** | **ARR (95% CI)** | ***p*-value** |
| Control |  | Ref. |  | Ref. |  | Ref. |  |
| Schizophrenia | Mental Illness | 2.9 (2.0-4.2) | <0.001 | 2.0 (1.3-2.9) | 0.001 | 2.8 (2.0-3.8) | <0.001 |
|  | + Treatment | 2.9 (1.9-4.4) | <0.001 | 1.9 (1.0-3.3) | 0.035 | 2.2 (1.4-3.6) | 0.001 |
|  | + Relapse | 2.9 (1.9-4.3) | <0.001 | 1.9 (1.2-3.2) | 0.011 | 2.6 (1.9-3.6) | <0.001 |
| Bipolar | Mental Illness | 3.0 (1.9-4.5) | <0.001 | 2.5 (1.9-3.3) | <0.001 | 3.1 (2.2-4.4) | <0.001 |
|  | + Treatment | 2.6 (1.9-3.6) | <0.001 | 1.8 (1.3-2.6) | <0.001 | 2.4 (1.7-3.3) | <0.001 |
|  | + Relapse | 2.7 (1.8-4.1) | <0.001 | 1.5 (1.0-2.3) | 0.03 | 2.4 (1.8-3.2) | <0.001 |
| Depression | Mental Illness | 2.7 (1.8-4.1) | <0.001 | 1.7 (1.2-2.3) | 0.002 | 2.6 (1.8-3.8) | <0.001 |
|  | + Treatment | 2.7 (1.8-4.1) | <0.001 | 1.8 (1.1-3.1) | 0.027 | 2.3 (1.6-3.6) | <0.001 |
|  | + Relapse | 3.0 (2.0-4.5) | <0.001 | 1.5 (0.8-2.5) | 0.179 | 2.2 (1.5-3.2) | <0.001 |

**Table F. Odds of perceiving stigmatizing beliefs of others, by treatment assignment, based on ordered logit regression (excluding 151 incorrectly reassigned participants).**

| **Perceived norms** | | **Most others unwilling for family member to marry** | | **Most others believe receiving divine punishment** | | **Most others believe Brings shame on family** | |
| --- | --- | --- | --- | --- | --- | --- | --- |
|  |  | **AOR (95% CI)** | ***p-value*** | **AOR (95% CI)** | ***p-value*** | **AOR (95% CI)** | ***p-value*** |
| Control | Ref. |  |  |  |  |  |  |
| Schizophrenia | Mental Illness | 14.0 (6.3-33.2) | 0.00 | 2.1 (1.5-2.9) | 0.00 | 3.8 (2.0-7.2) | 0.00 |
|  | + Treatment | 9.1 (3.5-24.3) | 0.00 | 2.7 (1.6-4.6) | 0.00 | 3.9 (1.9-7.8) | 0.00 |
|  | + Relapse | 19.0 (9.4-38.3) | 0.00 | 2.5 (1.7 -3.7) | 0.00 | 3.8 (2.5-5.7) | 0.00 |
| Bipolar | Mental Illness | 16.2 (6.9-38.3) | 0.00 | 3.1 (2.0-4.7) | 0.00 | 5.1 (4.0-6.4) | 0.00 |
|  | + Treatment | 10.5 (4.6-23.9) | 0.00 | 1.8 (1.3-2.5) | 0.00 | 3.3 (2.5-4.5) | 0.00 |
|  | + Relapse | 9.0 (4.0-20.5) | 0.00 | 2.0 (1.2-3.3) | 0.01 | 3.1 (1.8-5.1) | 0.00 |
| Depression | Mental Illness | 6.9 (2.5-19.1) | 0.00 | 2.8 (1.8-4.3) | 0.00 | 3.3 (1.9-5.8) | 0.00 |
|  | + Treatment | 10.9 (4.4-27.2) | 0.00 | 2.6 (1.8-3.7) | 0.00 | 3.1 (1.6-6.0) | 0.00 |
|  | + Relapse | 11.7 (5.3-25.6) | 0.00 | 1.7 (1.1-2.5) | 0.01 | 3.6 (2.4-5.4) | 0.00 |

*The estimated adjusted odds ratios in each cell refer to the relative odds of being in a higher category of perceiving that more people in their village (ranging from “very few, or no one” to “all or almost all”) hold the stigmatizing belief in question.*
